# A Palladium Catalyst System for the Efficient Cross-Coupling Reaction of Aryl Bromides and Chlorides with Phenylboronic Acid: Synthesis and Biological Activity Evaluation

**DOI:** 10.3390/molecules22030420

**Published:** 2017-03-07

**Authors:** Boubakri Lamia, Ahlem Chakchouk-Mtibaa, Bilel Hallouma, Lamjed Mansour, Lotfi Mellouli, Ismail Özdemir, Sedat Yaşar, Naceur Hamdi

**Affiliations:** 1 Research Laboratory of Environmental Sciences and Technologies (LR16ES09), Higher Institute of Environmental Sciences and Technology, University of Carthage, Hammam-Lif 2050, Tunisia; boubakri_lamia@yahoo.co.uk (B.L.); bilelhallouma@gmail.com (B.H.); 2 Laboratory of Microorganisms and Biomolecules of the Center of Biotechnology of Sfax-Tunisia, Road of Sidimansour, Km 6 B.P. 1117, Sfax 3018, Tunisia; ahlemchakchouk@yahoo.fr (A.C.-M.); lotfi.mallouli@cbs.rnrt.tn (L.M.); 3 Zoology Department, College of Science, King Saud University, P.O. Box 2455, Riyadh 11451, Saudi Arabia; lamjed.mansour@gmail.com; 4 İnönü University, Faculty of Science and Art, Department of Chemistry, Malatya 44280, Turkey; iozdemir44@gmail.com; 5 Chemistry Department, College of Science and Arts, Qassim University, Al-Rass 51921, Saudi Arabia

**Keywords:** *N*-heterocyclic carbene, palladium, cross-coupling reaction, biological activities

## Abstract

New benzimidazolium salts **1a**–**c** and their palladium bis-*N*-heterocyclic carbene complexes **2a**–**c** and palladium PEPPSI-type complexes **3a**–**c** were designed, synthesized and structurally characterized by NMR (^1^H and ^13^C), IR, DART-TOF mass spectrometry and elemental analysis. Then these complexes **2**–**3** were employed in the Suzuki-Miyaura cross-coupling reaction of substituted arenes with phenylboronic acid under mild conditions in toluene and DMF/H_2_O (1/1) to afford functionalized biaryl derivatives in good to excellent yields. The antibacterial activity of palladium bis-*N*-heterocyclic carbene complexes **2a**–**c** and palladium PEPPSI-type complexes **3a**–**c** was measured by disc diffusion method against Gram positive and Gram negative bacteria. Compounds **2a**, **2c** and **3a**–**c** exhibited potential antibacterial activity against four bacterial species among the five used indicator cells. The product **2b** inhibits the growth of the all five tested microorganisms. Moreover, the antioxidant activity determination of these complexes **2**–**3**, using 2.2-diphenyl-1-picrylhydrazyl (DPPH) as a reagent, showed that compounds **2a**–**c** and **3b** possess DPPH antiradical activity. The higher antioxidant activity was obtained from the product **2b** which has radical scavenging activity comparable to that of the two used positive controls (gallic acid “GA“ and tutylatedhydroxytoluene “BHT“). Investigation of the anti-acetylcholinesterase activity of the studied complexes showed that compounds **2b**, **3a**, and **3b** exhibited moderate activity at 100 μg/mL and product **2b** is the most active.

## 1. Introduction

*N*-heterocyclic carbene (NHC) ligands have become ubiquitous in the preparation of metal complexes with new catalytic applications. Mainly due to their applications in C-C bond formation reactions, a plethora of novel palladium-NHC complexes has been described, and a large number of review articles describing their chemistry have been published. In an attempt to provide a new vision of the topic, this article will focus our attention on the development of new palladium complexes with NHC ligands, paying special attention to their applications in catalytic processes other than the classical C-C coupling [1,2,3,4].

A wide range of NHC ligands which exhibit high activities in various important organic transformations when combined with metal pre-catalysts are now commercially available [5,6,7]. NHC imidazolidine ligands with sterically encumbering groups such as mesityl, 2,6-diisopropylphenyl, and adamantyl have been used in the Pd-catalyzed cyclization of anilides[8],amination of aryl chlorides [9], arylation with ester enolates to afford α-aryl esters [10]. Sonogashira reactions of unactivated alkyl bromides [11] and the ruthenium-catalyzed RCM reaction [12]. The coupling of aryl halides with organoboronic acids is one of the most important palladium-catalyzed cross-coupling reactions of both academic and industrial interest. In particular for the preparation of biaryl-containing molecules [13,14].

The reaction is the organic reaction of an aryl- or vinylboronic acid with an aryl or vinylhalide catalyzed by a palladium (0) complex. It is widely used to synthesize polyolefins, styrenes, and substituted biphenyls, and has been extended to incorporate alkyl bromides. Several reviews have been published [15,16,17]. However, the development of new ligands or the application of existing ligands in Suzuki reaction, particularly involving aryl chlorides as substrates, is still of considerable importance. In order to find more efficient palladium catalysts we have prepared a series of new (NHCs) stable NHC-PdCl_2_ pyridine complexes for the Suzuki coupling reaction.

Therefore, in this work, we describe the synthesis and characterization of new palladium (II) complexes. We also examined catalytic activities of these Pd (II) complexes **2**–**3** in the Suzuki-Miyaura cross-coupling reaction. The antibacterial, antioxidant and anti-acetylcholinesterase activities of the new synthesized complexes **2**–**3** were addressed as well.

## 2. Results and Discussion

### 2.1. Preparation of Benzimidazolium Salts ***1a**–**c***

**Scheme 1:**
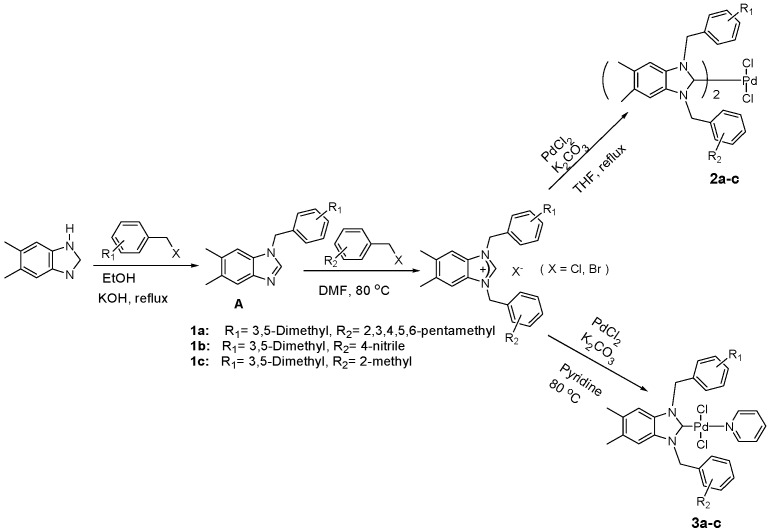
Synthesis of new benzimidazolium salts **1a**–**c** and their bis-NHC palladium complexes **2a**–**c** and PEPPSI-type complexes **3a**–**c**.

The precursors **1a**–**c** were prepared by the quaternization of the intermediate A with a variety of aryl chlorides or aryl bromides in DMF under 70 °C (Scheme 1). The benzimidazolium salts **1a**–**c** were obtained as white solids in very high to good yields of 95%, 90% and 79%, respectively. 

**Figure 1:**
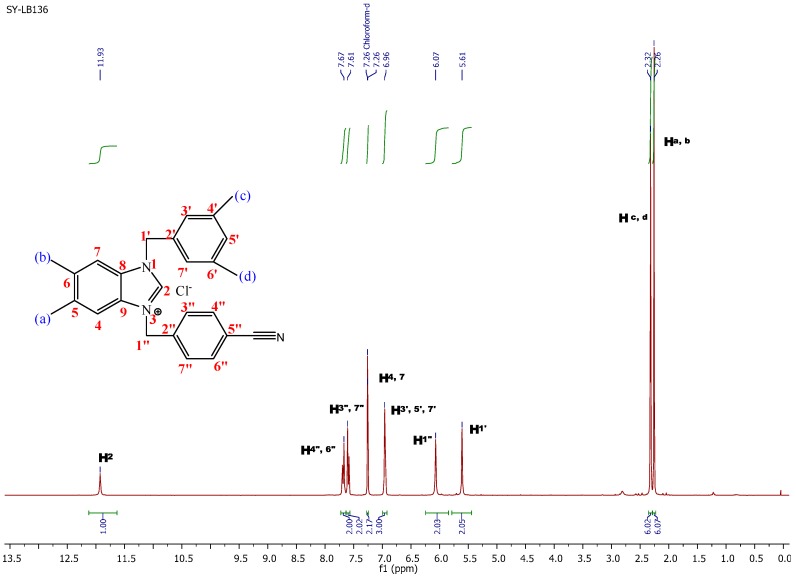
^1^H-NMR spectra of benzimidazolium salt **1b** in CDCl_3_.

Compounds **1a**–**c** were characterized by NMR (^1^H and ^13^C), IR, and DART-TOF mass spectrometry and elemental analysis. The ^1^H-NMR spectra of benzimidazolium salts **1a**–**c** were recorded in CDCl_3_. Here, the acidic proton signal of NCHN was seen as the most downfield signal and a sharp singlet at δ 10.91, 11.93 ppm and 11.84 was seen for **1a**, **1b** and **1c**, respectively (Figure 1).

The imino carbons (NCHN) were detected as typical singlets in the ^1^H decoupled mode at 141.7, 143.0, and 143.39 ppm. The IR data of **1a**, **1b** and **1c** clearly support the presence of the C-N group with ν(C-N) vibrations at 1545, 1570 and 1623 cm^−1^ respectively.

### 2.2. Preparation of bis-NHC-palladium Complexes ***2a**–**c*** and PEPPSI-type Complexes ***3a**–**c***

**Figure 2:**
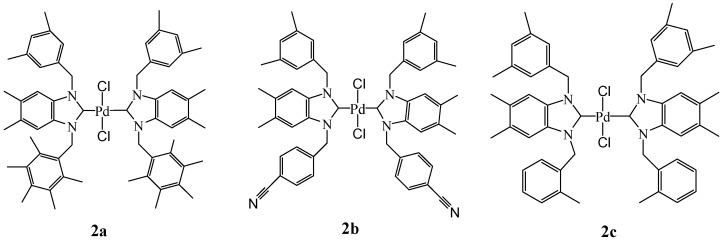
Structure of bis-NHC-palladium Complexes **2a**–**c**.

In order to obtain the PEPPSI-type complexes **3a**–**c**; we employed a reaction between PdCl_2_ and benzimidazolium salts **1a**–**c** in pyridine at 80 °C in the presence of K_2_CO_3_. Further, direct reaction of one equivalents of benzimidazolium salts **1a**–**c** with PdCl_2_ stirred at reflux in THF for 24 h in the presence of K_2_CO_3_ results in palladium complex formation **2a**–**c** (Figure 2).

**Figure 3:**
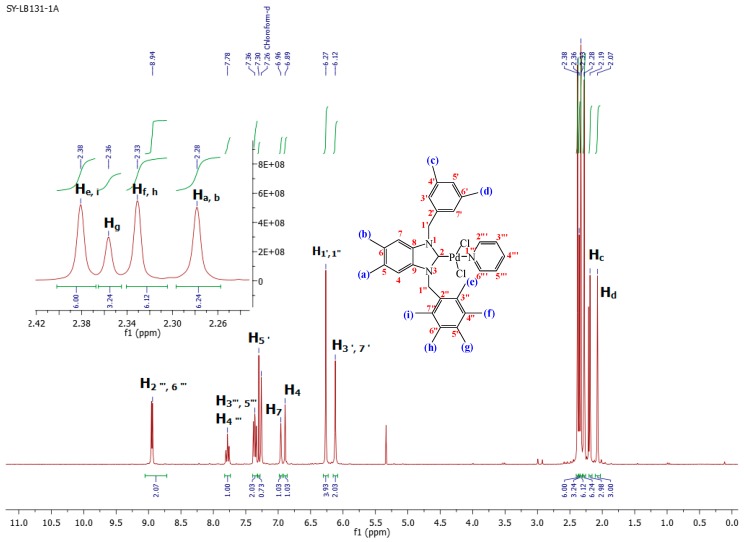
^1^H-NMR spectra of palladium PEPPSI-type complex **3a** in CDCl_3_.

Structural definitions of **2a**–**c** and **3a**–**c** were determined by NMR, IR spectroscopy, DART-TOF mass spectrometry and elemental analysis. The ^1^H-NMR spectra of compounds **2a**–**c** and **3a**–**c** were taken in CDCl_3_ at room temperature. In the ^1^H-NMR spectrum of **2a**, the aromatic protons appeared at between 6.28 and 7.24 ppm as a multiplet while methylic protons appeared between 1.98 and 2.42 ppm as singlets. In the ^1^H-NMR spectra of **3a**–**c**, (NCH_2_) was resonated at low fields δ 6.27, 6.24 and 6.08 respectively. While signals for the pyridine ring protons appeared between 7.36–8.94 ppm, 7.33–8.94 ppm and 7.28–8.91 ppm, respectively (Figure 3).

**Figure 4:**
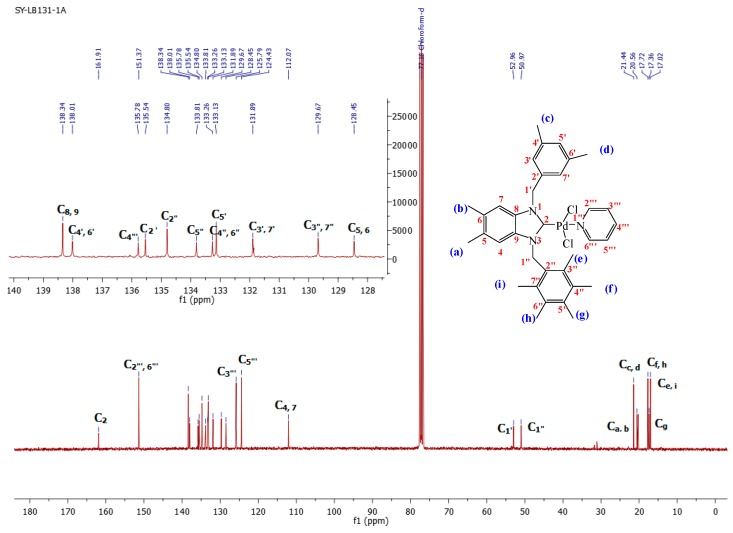
^13^C-NMR spectrum of palladium PEPPSI-type complex **3a** in CDCl_3_.

The absence of NCHN signal proton in a downfield for **2**–**3** indicated the successful formation of NHC complexes. The ^13^C-NMR spectra of complexes **2a**–**c** and **3a**–**c** were in good correlation with the structure of these compounds. ^13^C{^1^H} NMR spectra prove an increasing downfield shift of the NCN carbon from **1a**–**c** to **2a**–**c**: for example, the ^13^C{^1^H}N-C-N shifts of **1a** and **2a**, which are 141.7 and 180.8 ppm, respectively. The NCHN carbons for **3a**–**c** resonated at δ 161.9, 163.3 and 162.8 ppm respectively (Figure 4).

The functional groups of complexes **2**–**3** were identified by FT-IR spectroscopy. The IR(CN) band was observed at 1445 cm^−1^ for **2a**, 1462 cm^−1^ for **2b** and1463 cm^−1^ for **2c** in the FT-IR spectra. The same band shifted and appeared at 1461, 1463 and 1460 cm^−1^ for **3a**–**c**, respectively.

The contents of C, H, and N in palladium bis-*N*-heterocyclic carbene complexes **2a**–**c** and palladium PEPPSI-type complexes **3a**–**c** were determined by elemental analysis. The results agreed well with the theoretical formula of the complex.

**Figure 5:**
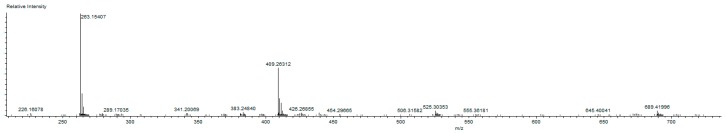
DART MS spectrum (DART-TOF-MS) of complex **3a**.

The obtained fragments are typical for each palladium bis-*N*-heterocyclic carbene complexes **2a**–**c** and palladium PEPPSI-type complexes **3a**–**c** and can provide further evidence for the characterization of the examined compounds. The MS spectrum of complex **3a** is given in Figure 5.

**Figure 6:**
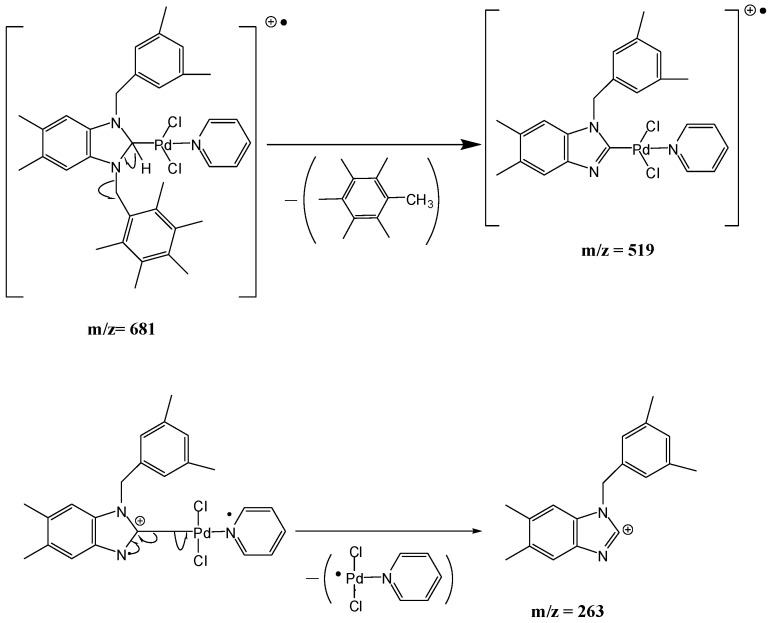
Mechanism of the fragmentation leading to the *m*/*z* = 263 peak.

The fragmentation leading to the *m*/*z* = 263 can occur via the mechanism of fragmentation given in Figure 6.

In order to demonstrate the utility of these NHC-PdCl_2_-pyridine complexes, we used them as co-catalysts in Suzuki-Miyaura cross-coupling reaction, which are common industry-applicable processes.

### 2.3. Suzuki Coupling Reaction of Aryl Chlorides/Bromides with Phenylboronic Acid

In a pilot study to examine the catalytic activity of bis NHC-palladium complexes **2a**–**c** and PEPPSI-type complexes **3a**–**c**, we initially tested the Suzuki cross coupling reaction between 4-chloroacetophenone and phenylboronic acid as a model reaction to determine optimum conditions. Here we compared both the effect of using toluene or DMF/H_2_O as the solvent, as well us using KOtBu or K_2_CO_3_ as the mineral base. As can be seen in Table 1, the best catalytic activities were only obtained when the Suzuki cross-coupling reaction was performed in DMF/H_2_O ratio was equal (1:1) with K_2_CO_3_ for PEPPSI complexes.

We tested the effect of common mineral bases such as K_2_CO_3_ and KO^t^Bu for the Suzuki coupling reactions of aryl chlorides. 1 (eq) of KO^t^Bu showed high performance in these catalytic systems. On the other hand, one can easily observe in Table 2 that a typical reaction of 4-chloroacetophenone and phenylboronic acid indicated that the reaction rate depended on the alkyl substituents. It can also be seen from Table 3 that the efficiency of complexes is not the same for each complex. For instance, the Suzuki cross-coupling reaction with catalyst **3**always afforded higher catalytic activity than that with catalyst **2**.

With the best conditions in hand, next we conducted further experiments to investigate the scope of the Suzuki cross-coupling reaction of catalysts **2** with various substrates, including aryl bromides and chlorides having electro *N*-withdrawing or electro *N*-donating substituents (Table 2). The highest conversion was up to 91% in the presence of KO^t^Bu within 6 h in toluene at 80 °C for catalyst **2b** with bromoanisole. On the other hands PEPPSI-type complexes afforded the efficient coupling of different aryl bromides and chloroacetophenone (Table 3), and in most cases the yield was higher than 90%, the reaction showed a good tolerance of different groups on the aromatic ring. 

When aryl chlorides were used as substrates, coupling products were formed with a lower yield (Table 2 and Table 3) chloroanisole and chlorotoluene (entry 4–9). This was expected on the basis of the higher values of the C-Cl bond energy with respect to C–Br. Nevertheless, good results were obtained for 4-chloroacetophenone.

### 2.4. Biological Activities

#### 2.4.1. Antibacterial Activity

The synthesized compounds palladium bis-*N*-heterocyclic carbene complexes **2a**–**c** and palladium PEPPSI-type complexes **3a**–**c** were evaluated in vitro for their antibacterial activity by the well diffusion method (Table 4). 

Globally, all complexes tested showed an important antibacterial activity against the three used Gram positive bacteria *Micrococcus luteus* LB 14110, *Staphylococcus aureus* ATCC 6538 and *Listeria monocytogenes* ATCC 19117. Concerning the activity against the two tested Gram negative microorganisms, all complexes inhibit the growth of *Salmonella Typhimurium* ATCC 14028 except the product **3c** and only the compound **2b** presents an inhibitory effect against *Pseudomonas aeruginosa* ATCC 49189 (Table 4).

In parallel, the Minimal Inhibitory Concentrations (MICs) values of palladium bis-*N*-heterocyclic carbene complexes **2a**–**c** and palladium PEPPSI-type complexes **3a**–**c** were determined against the two Gram positive bacteria *Micrococcus luteus* LB 14110 and *Listeria monocytogenes* ATCC 19117 and the Gram negative bacterium *Salmonella Typhimurium* ATCC 14028. The ampicillin was used as standard. As shown in Table 5, the MICs values range from 0.0197–0.625 mg/mL for *Micrococcus luteus* LB 14110; 0.078–1.25 mg/mL for *Listeria monocytogenes* ATCC 19117 and 1.25–5 mg/mL for *Salmonella Typhimurium* ATCC 14028. 

The most active compound was **2b** which presents against *Micrococcus luteus* LB 14110 the same MIC value of 0.0195 mg/mL than the used standard (ampicillin).The lowest MIC values of 0.0197  mg/mL were recorded for the Pd complexes **2b** against *Micrococcus luteus* LB 14110. The complex **2c** also have MIC values of 1.25 mg/mL against *Listeria monocytogenes* ATCC 19117. 

#### 2.4.2. DPPH Radical Scavenging

**Figure 7:**
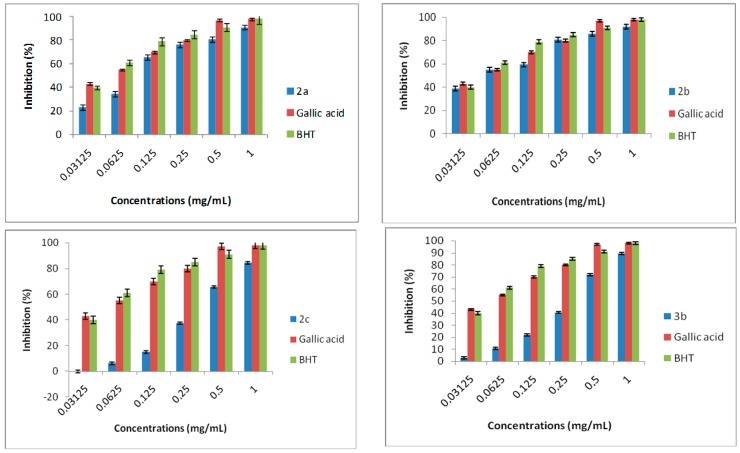
Scavenging activity of compounds **2a**–**c** and **3b** on DPPH radicals.

The hydroxyl radical is one of the most reactive products of reactive oxygen species (ROS). Among all free radicals, the hydroxyl radical is by far the most potent and therefore the most dangerous oxygen metabolite, which would result in cell membrane disintegration, membrane protein damage, DNA mutation and further initiate or propagate the development of many diseases. Elimination of this radical is one of the major aims of antioxidant administration [18]. Current research has shown that some antioxidants could act as the inducers of DNA damage response, which leads to cell death [19]. Therefore, in present study, we investigated whether the palladium bis-*N*-heterocyclic carbene complexes **2a**–**c** and palladium PEPPSI-type complexes **3a**–**c** could serve as a potent antioxidant. The scavenging activities of the complex on the DPPH radical were investigated. The radical was generated according to the method in the Experimental Section. From the results, we can see that, within the range of tested concentration, the average suppression ratios of DPPH increase along with the increase of the complex concentration (Figure 7). 

As shown in Figure 7 the compound **2b** showed higher antioxidant activity than other complexes. However the scavenging activity of the compound **2b** was very similar to that of the two used controlsbutylatedhydroxytoluene (BHT) and gallic acid (GA) known as good antioxidant compounds. No antioxidant activity was observed for the compounds **3a** and **3c**.

#### 2.4.3. Acetylcholinesterase Inhibition

The acetylcholinesterase enzyme (AChE) is an attractive target for the rational drug design and for the discovery of mechanism based inhibitors because of its role in the hydrolysis of the neurotransmitter acetylcholine (ACh). AChE inhibitors are the most effective approach to treat the cognitive symptoms of Alzheimer disease (AD) [20,21], and other possible therapeutic applications in the treatment of Parkinson′s disease, senile dementia, and ataxia, among others [22].

The results of AChEI of the synthesized compounds palladium bis-*N*-heterocyclic carbene complexes **2a**–**c** and palladium PEPPSI-type complexes **3a**–**c** are presented in Table 6. Three compounds **2b**, **3a**, and **3b** exhibited moderate AChEI activity at 100 μg/mL. As the antibacterial and antioxidant activities, the compound **2b** possesses the most active AChEI activity.

## 3. Experimental Section

### 3.1. General Information

All manipulations were performed using Standard Schlenck techniques under Argon atmosphere. Chemicals were purchased from Sigma Aldrich and used without further purification. All solvents were purified and dried by MBraun SPS 800 solvent purification system. Column chromatography was performed using silica gel 60 (70–230 mesh). ^1^H-NMR and ^13^C-NMR spectra were recorded at 300 MHz and 75 MHz, respectively. Chemical shifts, δ, are reported in ppm relative to the internal standard TMS for both ^1^H- and ^13^C-NMR. The products were characterized by GC (gas chromatography). Quantitative GC analyses were performed with a GC-2010 Plus gas chromatography (SHIMADZU). The NMR studies were carried out in high-quality 5 mm NMR tubes. Signals are quoted in parts per million as δ downfield from tetramethylsilane (δ = 0.00) as an internal standard. NMR multiplicities are abbreviated as follows: s = singlet, d = doublet, t = triplet, m = multiplet signal. IR spectra were recorded on a 398 spectrophotometer (Perkin-Elmer, King Saud University, Ryadh, Saudi Arabia). MS spectra were recorded on a ((DART-TOF-MS) instrument at King Saud University, Ryadh, Saudi Arabia). Elemental microanalysis was performed on an ElementarVario El III Carlo Erba 1108 elemental analyzer (INRAP, Sidi Thabet, Tunisia) and the values found were within ±0.3% of the theoretical values. Melting points were determined with Kofler bench at Isste of Borj Cedria (Hammam Lif, University of Carthage, Borj Cedria, Tunisia).

### 3.2. Synthesis of 1-(3,5-Dimethylbenzyl)-5,6-dimethylbenzimidazole *(**A**)*

To a solution of 5, 6-dimethylbenzimidazole (3 mmol, 4.38 g) resolved in 25 mL EtOH, (4 mmol, 2.5 g) of KOH was added and the reaction mixture was stirred for 15 min at room temperature. The corresponding aryl chlorides or bromides (3 mmol) were added slowly and the resulting mixture was stirred at room temperature for 1h and then heated for 8 h at 50 °C, after it was heated under reflux for 16 h. Solution was cooled to room temperature and the solvent was removed under reduced pressure. The yellow solid that formed was resolved with DCM (40 mL) and filtered. DCM was evaporated and the isolated product was characterized by NMR spectroscopy. Yield: 100(%). M.p. = 230 °C. FT-IR (KBr) ν, cm^−1^: 3065 (C-Harom); 1406 (C-N). ^1^H-NMR (CDCl_3_) δ (ppm):2.26 (s, 6H, Hc, d); 2.35 (s, 3H, Hb); 2.37 (s, 3H, Ha); 5.22 (s, 2H, H_1′_); 6.78 (s, 2H, H_3′,7′_); 6.93 (s, 1H, H_5′_); 7.08(s, 1H, H_7_); 7.58 (s, 1H, H_4_); 7.83 (s, 1H, H_2_).Anal. Calc. for C_9_H_11_N_2_: C, 73.437%; H, 7.532%; N, 19.031%, Found: C, 73.5; H, 7.6; N, 19.0%.

### 3.3. General Preparation of Benzimidazolium Salts ***1a**–**c***

To a solution of 5,6-dimethylbenzimidazole (3 mmol, 4.38 g) resolved in EtOH (25 mL) KOH (4 mmol, 2.5 g) was added and the reaction mixture was stirred for 15 min at room temperature. The corresponding aryl chlorides or bromides (3 mmol, 3equiv.) were added slowly and the resulting mixture was stirred at room temperature for 1h and then heated for 8 h at 50 °C, after it was heated under reflux for 16 h. Solution was cooled to room temperature and the solvent was removed under reduced pressure. The yellow solid that formed was resolved with DCM (40 mL) and filtered. DCM was evaporated and the isolated product was characterized by NMR spectroscopy.

A mixture of crude product (1 g) and corresponding aryl chlorides or bromides in DMF (2 mL) was stirred and heated at 70 °C for 1–2 days. The white solid that formed was washed with diethyl ether (30 mL), filtrated and dried under vacuum. 

*1*-*(3,5*-*Dimethylbenzyl)*-*5,6*-*dimethyl*-*3*-*(2,3,4,5,6*-*pentamethylbenzyl) benzimidazolium chloride* (**1a**). Yield: 95 (%). M.p. = 225 °C. FT-IR (KBr) ν, cm^−1^: 3055 (C-H_arom_); 1545 (C-N); ^1^H-NMR (CDCl_3_) δ (ppm): 2.23 (s, 15H, H_e,f,g,h,i_); 2.29 (s, 12H, H_a,b,c,d_); 5.77 (s, 2H, H_1′_); 5.79 (s, 2H, H_1′′_); 6.87 (s, 2H, H_3′,7′_); 6.91 (s, 1H, H_5′_); 7.08 (s, 1H, H_7_); 7.24 (s, 1H, H_4,_); 10.91 (s, 1H, H_2_). ^13^C-NMR (CDCl_3_) δ (ppm): 16.65 (Cg); 16.80 (C_f,h_); 16.99 (C_e,i_); 20.49 (C_c,d_); 20.90 (C_a,b_); 47.47 (C_1′′_); 50.87 (C_1′_); 113 (C_4,7_); 124.84 (C_5′_); 125.01 (C_4′,6′_); 129.89 (C_8,9_); 130.14 (C_4′′,6′′_); 132.95 (C_5′′_); 133.27 (C_5,6_); 133.59 (C_3′′,7′′_); 136.74 (C_2′_); 136.88 (C_2′′_); 138.51 (C_4′,6′_); 141.73 (C_2_). Anal. Calc. for C_30_H_38_N_2_Cl: C, 77.977%; H, 8.289%; N, 6.062%, Found: C, 78.1; H, 8.3; N, 6.1%.

*3*-*(4*-*Cyanobenzyl)*-*1*-*(3,5*-*dimethylbenzyl)*-*5,6*-*dimethyl*-*1H*-*benzo[d]imidazol*-*3*-*ium chloride* (**1b**). Yield: 90 (%). M.p. = 235 °C. FT-IR (KBr) ν, cm^−1^: 3062(C-H_arom_); 1570(C-N); ^1^H-NMR (CDCl_3_, δ (ppm): 2.26 (s, 6H, H_a,b_); 2.32 (s, 6H, H_c,d_); 5.61 (s, 2H, H_1′_); 6.07 (s, 2H, H_1′′_); 6.96 (s, 3H, H_3′,5′,7′_); 7.26 (s, 2H, H_4,7_); 7.61 (s, 2H, H_3′′,7′′_); 7.67 (s, 2H, H_4′′,6′′_); 11.93 (s, 1H, H_2_). ^13^C-NMR (CDCl_3_) δ (ppm): 20.81 (C_a,b_); 21.34 (C_c,d_); 50.46 (C_1′′_); 51.66 (C_1′_); 113.06 (C_5′′_); 113.58 (C_4,7_); 118.20 (CN); 125.64 (C_5′_); 129.10 (C_3′,7′_); 129.83 (C_7′′_); 129.93 (C_3′′_); 131.06 (C_8,9_); 132.42 (C_4′′,6′′_); 133.06 (C_5,6_); 137.74 (C_6′_); 137.82 (C_4′_); 138.52 (C_2′_); 139.28 (C_2′′_); 143.02 (C_2_). Anal. Calc. for C_26_H_27_N_3_Cl: C, 74.893%; H, 6.527%; N, 10.078%, Found: C, 74.9; H, 6.6; N, 10.1%.

*1*-*(3,5*-*Dimethylbenzyl)*-*5,6*-*dimethyl*-*3*-*(2*-*methylbenzyl)benzo*-*1H*-*imidazol*-*3*-*ium chloride* (**1c**). Yield: 79 (%). M.p. = 215 °C. FT-IR (KBr) ν, cm^−1^: 3064 (C-H_arom_); 1623(C-N); ^1^H-NMR (CDCl_3_) δ (ppm):2.26 (s, 9H, H_b,c,d_); 2.32 (s, 3H, H_a_); 2.40 (s, 3H, H_e_); 5.72 (s, 2H, H_1′_); 5.85 (s, 2H, H_1′′_); 6.94 (s, 1H, H_3′′_); 7.00 (s, 2H, H_3′,7′_); 7.03 (s, 1H, H_4′′_); 7.09 (s, 1H, H_5′′_); 7.14 (s, 1H, H_5′_);7.22 (s, 1H, H_7_); 7.24 (s, 1H, H_4_); 7.28 (s, 1H, H_6′′_); 11.84 (s, 1H, H_2_). ^13^C-NMR (CDCl_3_) δ (ppm):19.63 (C_a,b_); 20.79 (C_e_); 21.33 (C_c, d_); 50.02 (C_1′′_); 51.42 (C_1′_); 113.51 (C_4, 7_); 125.70 (C_5′′_); 126.77 (C_5′_); 127.88 (C_3′,7′_); 129.20 (C_4′′_); 130.02 (C_3′′_); 130.26 (C_6′′_); 130.86 (C_9_); 131.00 (C_8_); 131.36 (C_5,6_); 132.89 (C_2′_); 136.54 (C_7′′_); 137.35 (C_2′′_); 139.13 (C_4′,6′_); 143.39 (C_2_). Anal. Calc. for C_26_H_30_N_2_Cl: C, 76.919%; H, 7.448%; N, 6.900%, Found: C, 77.1; H, 7.5; N, 7.1%.

### 3.4. General Preparation of Palladium-bis-NHCs Complexes ***2a**–**c***

A Schlenk flask was charged with benzimidazolium salt (1 mmol), PdCl_2_ (0.5 mmol; 0.09 g), K_2_CO_3_ (0.6 g) and a stir bar under argon. Dried THF (25 mL) was then added as a solvent. The mixture was heated under reflux and stirred for 24 h at 100 °C. After completion, the reaction mixture was cooled at r.t. and the solvent was removed under vacuum. The solid formed was solubilized with DCM and purified by flash column, eluting with DCM until the product was completely recovered. DCM was removed under reduce pressure and the white solid was characterized by NMR spectroscopy. Further purification was done using recrystallization (DCM-hexane) or (DCM-CHCl_3_) to get pure complexes for analysis and catalysis.

*Bis-[1-(3,5-dimethylbenzyl)-5,6-dimethyl-3-(2,3,4,5,6–pentamethylbenzyl)benzimidazoliN-2-ylidene] palladium (IV) dichloride* (**2a**). Yield: 87 (%). M.p. = 233 °C. FT-IR (KBr) ν, cm^−1^: 3062 (C-Harom); 1445 (C-N); ^1^H-NMR (CDCl_3_) δ (ppm): 7.24 (s, 2H, H_14_, 14′, arom. CH); 7.07 (s, 2H, H_12_, 16, arom. CH); 6.92 (s, 1H, H_12′_, arom. CH); 6.83 (s, 1H, H_16′_, arom. CH); 6.73 (s, 2H, H_4,7_, arom. CH); 6.28 (s, 2H, H_4′_, 7′, arom. CH); 6.16 (s, 2H, H_17_, CH_2_); 6.02 (4H, H_10_, 10′, 2× CH_2_); 5.88 (2H, H_17′_, CH_2_); 2.42 (s, 6H, Hc, d, 2× CH_3_); 2.31 (s, 6H, Hc′, d′, 2× CH_3_); 2.28(s, 3H, Hg, CH_3_); 2.25 (s, 3H, Hg′, CH_3_); 2.22 (s, 12H, Ha, b, a′, b′, 4× CH_3_); 2.17 (s, 6H, He, i, 2× CH_3_); 2.15 (s, 12H, H_f,h,f′,h′_, 4× CH_3_); 1.98 (s, 6H, H_e′,i′_, 2× CH_3_). ^13^C-NMR (CDCl_3_) δ (ppm): 180.89 (NCN (C_2,2′_); 138.11–137.76 (arom. Cq (C_8,9,8′,9′_)); 136.09–135.90 (arom. Cq (C_13,15,13′,15′_); 135.34–135.12 (arom. Cq (C_11,11′_); 134.29 (arom. Cq (C_18′,18_); 133.16 (arom. Cq (C_21,21′_); 132.87–132.64 (arom. Cq (C_20,22,20′,22′_); 131.16 (arom. CH (C_14,14′_); 129.12–128.91 (arom. CH (C_12,16,12′,16′_); 125.41 (arom. Cq (C_19,23,19′,23′_); 125.18 (arom. Cq (C_5,6,5′,6′_); 112.37 (arom. CH (C_4,7,4′,7′_); 51.50 (CH_2_ (C_10,10′_); 50.86 (CH_2_ (C_17,17′_);16.82 (CH_3_ (C_e,i,e′,i′_); 17.13 (CH_3_ (C_f,h,f′,h′_); 17.57 (CH_3_ (C_g,g′_); 20.46 (CH_3_ (C_a,b,a,b′_); 21.05 (CH_3_ (C_c,d,c′,d′_). (DART-TOF-MS) = (*m*/*z* = 732.32). Anal. Calc. for C_60_H_74_N_4_PdCl_2_: C, 70.062%; H, 7.252%; N, 5.447%, Found: C, 70.1; H, 7.3; N, 5.6%.

*Bis-[3-(4-cyanolbenzyl)-1-(3,5-dimethylbenzyl)-5,6-dimethylbenzimidazoliN-2-ylidene] palladium (IV) dichloride* (**2b**). Yield: 88 (%). M.p. = 234 °C. FT-IR (KBr) ν, cm^−1^: 3060 (C-Harom); 1462 (C-N); ^1^H-NMR (CDCl_3_) δ (ppm): 7.52 (s, 2H, H _4,7_, arom. CH); 7.48 (s, 2H, H_4′,7′_, arom. CH); 7.41 (s, 4H, H_19,23,19′,23′_, arom. CH); 7.26 (s, 2H, H_14,14′_, arom. CH); 7.07 (s, 4H, H_12,16,12′,16′_, arom. CH); 6.95 (s, 2H, H_20,22_, arom. CH); 6.85 (s, 2H, H_20′,22′_, arom. CH); 5.97 (s, 2H, H_10_, CH_2_); 5.90 (s, 2H, H_17_, CH_2_); 5.82 (s, 2H, H_17′_, CH_2_); 5.76 (s, 2H, H_10′_, CH_2_); 2.24 (s, 12H, H_c,d,c′,d′_, 4× CH_3_); 2.21–2.20 (s, 12H, Ha, b, a′, b′, 4× CH_3_). ^13^C-NMR (CDCl_3_) δ (ppm): 181.34 (NCN (C_2,2′_); 141.95 (arom. Cq (C_8,9,8′,9′_); 138.80 (arom. Cq (C_18,18′_); 136.06 (arom. Cq (C_13,15,13′,15′_); 133.47 (arom. Cq (C_11,11′_); 133.19 (arom. CH (C_20,22_); 133.00 (arom. CH (C_20′,22′_); 132.02 (arom. CH (C_14,14′_); 130.03–129.89 (arom. CH (C_19,23,19′,23′_); 128.44 (arom. CH (C_12,16,12′,16′_); 125.78–125.47 (arom. Cq (C_5,6,5′,6′_); 119.21 (CN); 112.21 (arom. CH (C_4,7,4′_,_7′_); 111.90 (arom. Cq (C_21,21′_); 52.08 (CH_2_ (C_10,10′_); 51.46 (CH_2_ (C_17,17′_); 21.76 (CH_3_ (C_c,d,c′,d′_); 20.83 (CH_3_ (C_a_,_b_,_a′_,_b′_). (DART-TOF-MS) (*m*/*z* = 642.32). Anal. Calc. for C_52_H_52_N_6_PdCl_2_: C, 66.560%; H, 5.586%; N, 8.956%, Found: C, 66.6; H, 5.6; N, 8.9%.

*Bis-[1-(3,5-dimethylbenzyl)-5,6-dimethyl-3-(2-methylbenzyl)benzimidazoliN-2-ylidene] palladium (IV) dichloride* (**2c**). Yield: 95 (%). M.p. = 245 °C. FT-IR (KBr) ν, cm^−1^: 3064 (C-Harom); 1463 (C-N); ^1^H-NMR (CDCl_3_) δ (ppm): 7.14 (s, 4H, H_22,23,22′,23′_, arom. CH); 7.09 (s, 4H, H_14,14′,21,21′_, arom. CH); 7.05 (s, 4H, H_12,16,12′,16′_, arom. CH); 6.94 (s, 1H, H_4_, arom. CH); 6.90 (s, 1H, H_7_, arom. CH); 6.84 (s, 1H, H_4′_, arom. CH); 6.81 (s, 1H, H_7′_, arom. CH); 6.73 (s, 1H, H_20_, arom. CH); 6.71 (s, 1H, H_20′_, arom. CH); 5.90 (s, 2H, H_10_, CH_2_); 5.84 (s, 2H, H_10′_, CH_2_); 5.75 (s, 2H, H_17_, CH_2_); 5.72 (s, 2H, H_17′_, CH_2_); 2.22 (s, 6H, Hc, d, 2× CH_3_); 2.21(s, 6H, H_c′,d′_, 2× CH_3_); 2.18 (s, 12H, H_a,b,a′,b′_, 4× CH_3_); 2.15 (s, 6H, H_e,e′_, 4× CH_3_). ^13^C-NMR (CDCl_3_) δ (ppm): 181.91 (NCN (C_2,2′_); 138.55 (arom. Cq (C_8,9,8′,9′_); 136.50 (arom. Cq (C_13,15,13′,15′_); 135.63 (arom. Cq (C_11,11′_); 135.48 (arom. Cq (C_19′,19_); 134.69–134.51 (arom. Cq (C_18,18′_); 133.85–133.77 (arom. CH (C_20,20′_); 133.61–133.50 (arom. CH (C_14,14′_); 132.45 (arom. CH (C_21_,_21′_); 130.41 (arom. CH (C_12,16_); 129.71 (arom. CH (C_12′,16′_); 127.90–127.67 (arom. CH (C_23,23′_); 126.86 (arom. Cq (C_5,6_); 126.76 (arom. Cq (C_5′,6′_); 125.98–125.73 (arom. CH (C_22,22′_); 111.99–111.63 (arom. CH (C_4,7,4′,7′_); 52.20 (CH_2_ (C_10,10′_); 49.96 (CH_2_ (C_17,17′_); 21.70 (CH_3_ (C_c,d,c′,d′_); 20.37 (CH_3_ (C_e,e′_); 19.79 (CH_3_ (C_a,b,a′,b′_). (DART-TOF-MS) (*m*/*z* = 383.2, *m*/*z* = 367.29). Anal. Calc. for C_52_H_58_N_9_PdCl_2_: C, 63.317%; H, 5.927%; N, 12.780%, Found: C, 63.4; H, 5.9; N, 12.8%.

### 3.5. General Preparation of PEPPSI Complexes ***3a**–**c***

A pressure tube was charged with benzimidazolium salts (1 mmol), PdCl_2_ (1 mmol; 0.18 g), K_2_CO_3_ (0.6 g) and a stir bar under atmosphere. Pyridine (1 mmol, 3 mL) was then added as the solvent and the reactant. The mixture was heated and stirred for 16 h at 80 °C. After cooling to r.t, the reaction mixture was diluted with CH_2_Cl_2_ and purified by flash column, eluting with DCM until the product was completely recovered. DCM was evaporated and the crude product was washed with 3 × 20 mL hexane. The yellow solid was characterized by NMR spectroscopy. Further purification was done using recrystallization (DCM-hexane) to get pure complexes for analysis and catalysis.

*1*-*(3,5*-*Dimethylbenzyl)*-*5,6*-*dimethyl*-*3*-*(pentamethylbenzyl)-benzimidazoliN*-*2*-*ylidene*-*N*-*(pyridine)dichloro palladium (II) complex* (**3a**):Yield: 92(%). M.p. = 215 °C. FT-IR (KBr) ν, cm^−1^: 3062 (C-Harom); 1461 (C-N); ^1^H-NMR (CDCl_3_) δ (ppm): 8.94 (dd, 2H, (arom. CH (C_2′′′,6′′′_); 7.78 (m, 1H, (arom. CH (H_4′′′_)); 7.36 (m, 2H, (arom. CH (H_3′′′,5′′′_); 7.30 (s, 1H, (arom. CH (H_5′_); 6.96 (s, 1H, (arom.CH (H_7_); 6.89 (s, 1H, (arom. CH (H_4_); 6.27 (s, 4H, (2× CH_2_ (H_1′,1′′_); 6.12 (s, 2H, (arom. CH (H_3′,7′_); 2.38 (s, 6H, 2× CH_3_ (He, i); 2.36 (s, 3H, (CH_3_ (H_g_); 2.33 (s, 6H, (2× CH_3_ (H_f,h_); 2.28 (s, 6H, (2× CH_3_ (H_a, b_); 2.19 (s, 3H, (CH_3_ (Hc); 2.07 (s, 3H, (CH_3_ (Hd). ^13^C-NMR (CDCl_3_) δ (ppm): 161.91 (NCN (C_2_); 151.37 (arom. CH (C_2′′′,6′′′_); 138.34 (arom. Cq (C_8,9_); 138.01 (arom. Cq (C_4′,6′_); 135.78 (arom. CH (C_4′′′_); 135.54 (arom. Cq (C_2′_); 134.80 (arom. Cq (C_2′′_); 133.81 (arom. Cq (C_5′′_); 133.26 (arom. Cq (C_4′′,6′′_); 133.13 (arom. CH (C_5′_); 131.89 (arom. CH (C_3′,7′_); 129.67 (arom. Cq (C_3′′,7′′_); 128.45 (arom. Cq (C_5,6_); 125.79–124.43 (arom. CH (C_3′′′,5′′′_); 112.07 (arom. CH (C_4,7_); 52.96 (CH_2_ (C_1′_); 50.97 (CH_2_ (C_1′′_); 21.44 (CH_3_ (C_c_,_d_); 20.56 (CH_3_ (C_a,b_); 17.32 (CH_3_ (C_f,h_); 17.36 (CH_3_ (C_g_); 17.02 (CH_3_ (C_e,i_). (DART-TOF-MS) (*m*/*z* = 520.3). Anal. Calc. for C_35_H_42_N_3_PdCl_2_: C, 61.634%; H, 6.207%; N, 6.161%, Found: C, 61.7; H, 6.3; N, 6.2%.

*1*-*(3,5*-*Dimethylbenzyl)*-*3*-*(cyanobenzyl)*-*5,6*-*dimethylbenzimidazol*-*2*-*ylidene*-*N*-*(pyridine)dichloro palladium (II) complex* (**3b**). Yield: 85(%). M.p. = 225 °C. FT-IR (KBr) ν, cm^−1^: 3062 (C-Harom); 1463 (C-N); ^1^H-NMR (CDCl_3_) δ (ppm): 8.94 (d, 2H, H_2′′′,6′′′_, arom. CH); 7.76 (m, 1H, H_4′′′_, arom. CH); 7.66 (s, 4H, H_3′′,4′′,6′′,7′′_, arom. CH); 7.33 (m, 2H, H_3′′′,5′′′_, arom. CH); 7.24 (s, 1H, H_4,7_, arom. CH); 6.96(s, 1H, H_3′,7′_, arom. CH); 6.78 (s, 1H, H_5′_, arom. CH); 6.24 (s, 2H, H_1′_, CH_2_); 6.08 (s, 2H, H_1′′_, CH_2_); 2.30 (s, 6H, H_c,d_, 2× CH_3_); 2.22 (s, 6H, H_a,b_, 2× CH_3_). ^13^C-NMR (CDCl_3_) δ (ppm): 163.36 (NCN (C_2_); 151.37 (arom. CH (C_2′′′_,_6′′′_); 140.94 (arom. Cq (C_8,9_); 138.57 (arom. Cq (C_2′′_); 138.32 (arom. Cq (C_4′_,_6′_); 135.10 (arom. CH (C_4′′′_); 133.37 (arom. Cq (C_2′_); 133.01 (arom. CH (C_4′′_,_6′′_); 132.83 (arom. CH (C_5′_); 129.96 (arom. CH (C_3′′_,_7′′_); 128.53 (arom. CH (C_3′_,_7′_); 125.79 (arom. Cq (C_5,6_); 124.64 (arom. CH (C_3′′′,5′′′_); 118.77 (CN); 112.05 (arom. CH (C_4,7_); 111.10 (arom. Cq (C_5′′_); 52.91 (CH_2_ (C_1′_); 52.25 (CH_2_ (C_1′′_); 21.44 (CH_3_ (C_c,d_); 20.40 (CH_3_ (C_a,b_). (DART-TOF-MS) (*m*/*z* = 523.3, *m*/*z* = 263.1). Anal. Calc. for C_31_H_31_N_4_PdCl_2_: C, 58.458 %; H, 4.906 %; N, 8.796 %, Found: C, 58.7; H, 5.1; N, 8.8%.

*1*-*(3,5*-*Dimethylbenzyl)*-*5,6*-*dimethyl*-*3*-*(2*-*methylbenzyl)*-*benzimidazoli*-*2*-*yl*-*idene*-*N*-*(pyridine)dichloro palladium* *(II) complex*(**3c**). Yield: 90(%). M.p. = 235 °C. FT-IR (KBr) ν, cm^−1^: 3061 (C-Harom); 1460 (C-N); ^1^H-NMR (CDCl_3_) δ (ppm): 8.91 (s, 2H, H_2′′′,6′′′_, arom. CH), 7.71 (t, 1H, H_4′′′_, arom. CH); 7.28 (m, 2H, H_3′′′,5′′′_, arom. CH); 7.24 (s, 1H, H_3′′_, arom. CH); 7.22 (s, 3H, H_4′′,5′′,6′′_, arom. CH); 7.20 (s, 1H, H_5′_, arom. CH); 6.92 (s, 2H, H_3′,7′_, arom. CH); 6.71 (s, 2H, H_4,7_, arom. CH); 6.13 (s, 2H, H_1′_, CH_2_); 6.08 (s, 2H, H_1′′_, CH_2_); 2.49 (s, 3H, H_e_, CH_3_); 2.28(s, 6H, H_c,d_, 2× CH_3_); 2.19 (s, 3H, H_a_, CH_3_); 2.16 (s, 3H, H_b_, CH_3_). ^13^C-NMR (CDCl_3_) δ (ppm): 162.84 (NCN (C_2_); 151.44 (arom. CH (C_2′′′,6′′′_); 138.46 (arom. Cq (C_8,9_); 138.10 (arom. Cq (C_4′,6′_); 135.53 (arom. CH (C_4′′′_); 133.50 (arom .Cq (C_2′_); 133.26 (arom. Cq (C_2′′_); 132.55 (arom. Cq (C_7′′_); 130.45 (arom. CH (C_6′′_); 129.80 (arom. CH (C_5′_); 128.12 (arom. CH (C_5′′_); 127.92 (arom. CH (C_3′,7′_); 126.61 (arom. CH (C_3′′_); 125.76 (arom. Cq (C_5,6_); 124.49 (arom. CH (C_4′′_); 111.77 (arom. CH (C_3′′′,5′′′_); 111.49 (arom. CH (C_4,7_); 52.88 (CH_2_ (C_1′_); 50.30 (CH_2_ (C_1′′_); 21.44 (CH_3_ (C_c,d_); 20.41(CH_3_ (Ce); 19.89 (CH_3_ (C_a,b_). (DART-TOF-MS) (*m*/*z* = 464.26, *m*/*z* = 384.2). Anal. Calc. for C_31_H_34_N_3_PdCl_2_: C, 59.483%; H, 5.475 %; N, 6.713%, Found: C, 59.5; H, 5.6; N, 6.8%.

### 3.6. General Procedure for the Suzuki Miyaura Reaction 

Phenylboronic acid (0.75 mmol), aryl halides (0.5 mmol), palladium catalyst (0.25 mol %), base (1 mmol) and solvent (1:1) (6 mL) were added under argon to a Schlenk flask containing a magnetic stir bar. The mixture was vigorously stirred at 80 °C for the indicate time. Upon completion, the mixture was cooled to room temperature, extracted with ethyl acetate (5 mL) and filtered through a short pad of silica gel. The filtrate was sampled at intervals for GC analysis.

### 3.7. Antibacterial Activity

#### 3.7.1. Bacterial Strains, Media and Growth Conditions

Bacteria strains, Gram-positive bacteria: *Micrococcus luteus* LB 14110, *Staphylococcus aureus* ATCC 6538 and *Listeria monocytogenes* ATCC 19117, and Gram-negative bacteria: *Salmonella Typhimurium* ATCC 14028 and *Pseudomonas aeruginosa* ATCC 49189, used as indicator microorganisms for the antibacterial activity assays, were obtained from International Culture Collections (ATCC) and local culture collection of Laboratory of Microorganisms and Biomolecules of the Centre of Biotechnology of Sfax-Tunisia. For antibacterial determination, indicator microorganisms were grown overnight in Luria-Bertani (LB) agar medium composed of (g/L): peptone 10; yeast extract 5; and NaCl 5 at pH 7.2 under aerobic conditions and constant agitation (200 rpm) at 30 °C for *M. luteus* LB14110, and *L. monocytogenes* ATCC 19117 and at 37 °C for *S. aureus* ATCC 6538, S. *Typhimurium* ATCC 14028 and *P. aeruginosa* ATCC 49189, and then diluted 1:100 in LB media and incubated for 5 h under constant agitation (200 rpm) at the appropriate temperature. 

#### 3.7.2. Agar Well Diffusion Method

Agar well diffusion method was employed for the determination of the antibacterial activity of the synthesized compounds with some modifications according to [23].

#### 3.7.3. MIC Determination

The antimicrobial activities of the synthesized compounds were determined by the minimum inhibitory concentration (MICs) in accordance with NCCLS guideline M7-A_6_ and M38-P [24]. The test was performed in sterile 96-well microplates with a final volume in each microplate well of 100 μL. The synthesized compounds (20 mg/mL) were properly prepared in solution of dimethylsulfoxide (DMSO)/water (1/9; *v*/*v*). The inhibitory activity of each synthesized compound was transferred to each well in order to obtain a twofold serial dilution of the original sample and to produce the concentration range of 0.0048–20 mg/mL.

### 3.8. DPPH Radical Scavenging Activity 

DPPH possess a proton free radical, when DPPH encounters proton radical scavengers its purple color fades rapidly. This assay determines the scavenging of stable radical species according to the method of [25], with slight modifications. Briefly, synthesized compounds were dissolved in dimethylsulfoxide (DMSO)/water (1/9; *v*/*v*) and diluted with ultrapure water at different concentrations (1, 0.5, 0.250, 0.125, 0.0625, 0.03125 mg/mL). Then, 500 μL of a 4% (*w*/*v*) solution of DPPH radical in methanol was mixed with 500 μL of samples. The mixture was incubated for 30 min in the dark at room temperature. The scavenging capacity was determined spectrophotometrically by monitoring the decrease in absorbance at 517 nm against a blank. The percentage of antiradical activity (% ArA) had been calculated as follows: % ArA = [(absorbance of control − absorbance of test sample)/absorbance of control] × 100. All tests are assayed in triplicate and expressed as the average ± standard deviation of the measurements.

### 3.9. Acetylcholinesterase Inhibitory Potential

AChE inhibitory activity was measured by slightly modified spectrophotometric method of Ellman et al.[26]. Electric eel AChE was used, while acetylthiocholine iodide (ATCI) was employed as substrate of the reaction. 5.5′-dithiobis-(2-nitrobenzoic acid) (DTNB) was used for the measurement of the antiacetylcholinesterase activity. Briefly, in this method, 100 μL of Tris buffer at 50 mM (pH 8.0), 30 μL of sample or standard and 5 μL of AChE enzyme (0.5 U/mL) were added in a 96 well microplate and incubated for 10 min at 25 °C. Then, 142 μL of DTNB (3 mM) and 23 μL of substrate (75 mM) were added. Percentage of inhibition of AChE was determined by comparison of rates reaction of samples relative to control (10% DMSO in Tris buffer) using the following formula:

% AChEI = 1 − (δA sample/δA control) × 100

where δA sample: Sample absorbance at zero time − Sample absorbance at the end of reaction, and δA control: Control absorbance at zero time − Control absorbance at the end of reaction. Galanthamine, an antiacetylcholinesterase alkaloid type of drug obtained from the snowdrop bulbs (*Galanthus* sp.), was used as standard. All synthesized compounds have been tested at 100 μg/mL of concentration. This determination was done in triplicate and obtained results were very similar. The reported value is the average of the three tests.

## 4. Conclusions

In summary, a simple route for the synthesis of palladium (II) complexes containing N donor ligands has been successfully demonstrated and the products fully characterized by NMR, IR, DART-TOF mass spectrometry and elemental analysis. These air and moisture stable palladium (II) complexes efficiently catalyze the cross-coupling of aryl bromides and chlorides (from electron rich to electron poor) with phenylboronic acid in DMF/H_2_O at 80 °C for 24 h, using KO^t^Bu or K_2_CO_3_ as bases, without addition of free ligand or any promoting additive, no significant homocoupling of phenylboronic acid to unsubstituted biphenyl was observed. 

The obtained complexes **2a**–**c** and **3a**–**c** were tested for their antibacterial activity against *Micrococcus luteus* LB 14110, *Staphylococcus aureus* ATCC 6538, *Listeria monocytogenes* ATCC 19117, *Salmonella* *Typhimurium* ATCC 14028 and *Pseudomonas aeruginosa* ATCC 49189. Obtained results show that the obtained complexes **2a**–**c** and **3a**–**c** have an effective antibacterial activity against the used indicator bacteria. However, it should be noted that the product **2b** strongly inhibits the growth of the all tested food-borne pathogens and clinical microorganisms. Interestingly, this compound **2b**, possesses scavenging activity very similar to that of the two well-known antioxidant standards butylatedhydroxytoluene (BHT) and gallic acid (GA). Three compounds **2b**, **3a**, and **3b** exhibited moderate AChEI activity and the product **2b** was the most active, with an acetylcholinesterase inhibitory activity of 38.15% at 100 μg/mL. Though the complexes showed slightly more antibacterial activities than other reported complexes, their strong abilities to bind with DNA and scavenge free radicals compared to other reported palladium complexes was notable [27,28,29,30].

## Figures and Tables

**Table 1:**
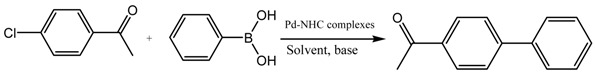
Effect of solvent and base on Suzuki cross-coupling reaction ^a^.

Entry	Pd-NHC Complexes	Solvent	Base	Yield (%) ^b^
1	**2a**	Toluene	KO^t^Bu	73
2	**3a**			60
4	**2a**	DMF/H_2_O	KO^t^Bu	0
5	**3a**			89
6	**2a**	DMF/H_2_O	K_2_CO_3_	1
7	**3a**			90

^a^ *Reaction conditions*: Phenylboronic acid (0.75 mmol), 4-chloroacetophenone (0.5 mmol), Pd-NHC complexes (0.25 mol %),base (1 mmol), 6 mL solvent (1:1), 80 °C, 3 h. Under Argon; ^b^ Conversions were determined by GC.

**Table 2:**
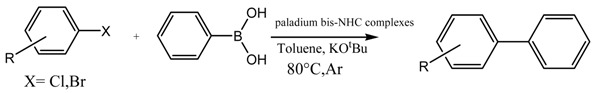
The Suzuki coupling reaction of aryl chlorides/bromides with phenylboronic acid catalyzed by different unsymmetrical palladium-bis-NHCs complexes ^a^.

Entry	Ar-X	Pd-NHC Complexes	Time (h)	Yield (%) ^b^
1	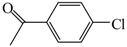	**2a**	3	73
2		**2b**	3	66
3		**2c**	3	83
4	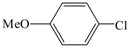	**2a**	12	6
5		**2b**	12	4
6		**2c**	12	5
7	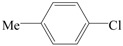	**2a**	12	6
8		**2b**	12	2
9		**2c**	12	2
10	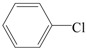	**2a**	12	28
11		**2b**	12	25
12		**2c**	12	20
13	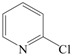	**2a**	12	4
14		**2b**	12	14
15		**2c**	12	14
16	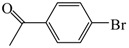	**2a**	3	85
17		**2b**	3	90
18		**2c**	3	91
19	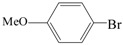	**2a**	6	76
20		**2b**	6	91
21		**2c**	6	84
22	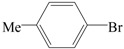	**2a**	6	47
23		**2b**	6	75
24		**2c**	6	85
25	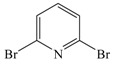	**2a**	6	Mono = 42 Di = 58
26		**2b**	6	Mono = 44 Di = 56
27		**2c**	6	Mono = 25 Di = 75

Mono: monoarylated; Di: diarylated; ^a^ *Reaction conditions*: Phenylboronic acid (0.75 mmol), aryl halides (0.5 mmol), Pd-NHC complexes (0.25 mol %), KO^t^Bu (1 mmol), 6 mL Toluene, 80 °C. Under Argon; ^b^ Conversions were determined by GC.

**Table 3:**
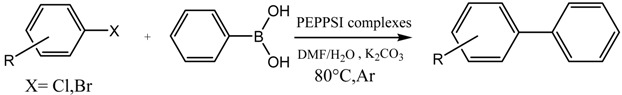
The Suzuki Coupling Reaction of Aryl Chlorides/Bromides with phenylboronic Acid catalyzed by different unsymmetrical PEPPSI complexes ^a^.

Entry	Ar-X	Pd-NHC Complexes	Time (h)	Yield (%) ^b^
1	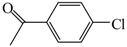	**3a**	3	90
2		**3b**	3	100
3		**3c**	3	99
4	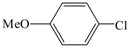	**3a**	12	28
5		**3b**	12	9
6		**3c**	12	25
7	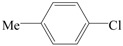	**3a**	12	34
8		**3b**	12	15
9		**3c**	12	22
10	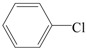	**3a**	12	77
11		**3b**	12	67
12		**3c**	12	67
13	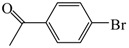	**3a**	3	100
14		**3b**	3	100
15		**3c**	3	100
16	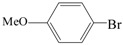	**3a**	6	100
17		**3b**	6	100
18		**3c**	6	100
19	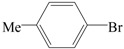	**3a**	6	100
20		**3b**	6	100
21		**3c**	6	100
22	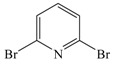	**3a**	6	100 Diarylated
23		**3b**	6	100 Diarylated
45		**3c**	6	100 Diarylated

^a^ *Reaction conditions*: Phenylboronic acid (0.75 mmol), aryl halides (0.5 mmol), Pd-NHC complexes (0.25 mol %), K_2_CO_3_(1 mmol), 6 mL DMF/H_2_O (1:1), 80 °C. Under Argon; ^b^ Conversions were determined by GC.

**Table 4:** Antibacterial activity of the synthesized palladium bis-*N*-heterocyclic carbene complexes (**2a**–**c**) and Palladium PEPPSI-type complexes (**3a**–**c**).

Microorganism Indicator	Compounds	Inhibition Zone (mm)
*Micrococcus luteus* LB 14110	**2a**	18 ± 0.5
	**2b**	23 ± 0.2
	**2c**	24 ± 0.1
	**3a**	25 ± 0.3
	**3b**	30 ± 0.5
	**3c**	22 ± 0.4
*Staphylococcus aureus* ATCC 6538	**2a**	16 ± 1.1
	**2b**	17 ± 0.5
	**2c**	15 ± 0.3
	**3a**	15 ± 0.3
	**3b**	16 ± 0.5
	**3c**	12 ± 0.4
*Listeria monocytogenes* ATCC 19117	**2a**	20 ± 0.4
	**2b**	16 ± 1.5
	**2c**	19 ± 0.5
	**3a**	16 ± 0.3
	**3b**	16 ± 0.3
	**3c**	14 ± 0.5
*Salmonella Typhimurium* ATCC 14028	**2a**	14 ± 0.4
	**2b**	16 ± 0.4
	**2c**	13 ± 0.3
	**3a**	12 ± 0.1
	**3b**	16 ± 0.5
	**3c**	-
*Pseudomonas aeruginosa* ATCC 49189	**2a**	-
	**2b**	16 ± 0.2
	**2c**	-
	**3a**	-
	**3b**	-
	**3c**	-

**Table 5:** Minimum Inhibitory Concentrations (MICs) expressed in mg/ml of compounds **2**–**3**.

Microorganism Indicator	Compounds	MIC (mg/mL)
*Micrococcus luteus* LB 14110	**2a**	0.039
	**2b**	0.0197
	**2c**	0.025
	**3a**	0.3125
	**3b**	0.039
	**3c**	0.625
	Ampicillin	0.0195
*Listeria monocytogenes* ATCC 19117	**2a**	1.25
	**2b**	0.078
	**2c**	1.25
	**3a**	2.5
	**3b**	0.3125
	**3c**	1.25
	Ampicillin	0.039
*Salmonella typhimurium* ATCC14028	**2a**	2.5
	**2b**	1.25
	**2c**	2.5
	**3a**	2.5
	**3b**	2.5
	**3c**	5
	Ampicillin	0.625

**Table 6:** Acetylcholinesterase inhibitory activity (AChEI) (%) of compounds **2a**–**c** and **3a**–**c**.

Compounds	(AChEI) (%)
**2a**	—
**2b**	38.15
**2c**	—
**3a**	32.15
**3b**	32.80
**3c**	—
